# The feasibility of using haptic devices to engage people with chronic traumatic brain injury in virtual 3D functional tasks

**DOI:** 10.1186/1743-0003-11-117

**Published:** 2014-08-08

**Authors:** Lynn H Gerber, Cody G Narber, Nalini Vishnoi, Sidney L Johnson, Leighton Chan, Zoran Duric

**Affiliations:** Center for the Study of Chronic Illness and Disability, George Mason University, 4400 University Dr., 22030 Fairfax, VA USA; Department of Computer Science, George Mason University, 4400 University Dr., 22030 Fairfax, VA USA; Rehabilitation Medicine Department, Clinical Center, NIH, 9000 Rockville Pike, 20892 Bethesda, MD USA

**Keywords:** Traumatic brain injury, Virtual reality, Haptic devices, Fine motor, Cognitive performance

## Abstract

**Background:**

The primary aim of this study was to assess the level of engagement in computer-based simulations of functional tasks, using a haptic device for people with chronic traumatic brain injury. The objectives were to design functional tasks using force feedback device and determine if it could measure motor performance improvement.

**Methods:**

A prospective crosssectional study was performed in a biomedical research facility. The testing environment consisted of a single, interactive, stylus-driven computer session navigating virtual scenes in 3D space. Subjects had a haptic training session (TRAIN) and then had three chances to perform each virtual task: (i) remove tools from a workbench (TOOL), (ii) compose 3 letter words (SPELL), (iii) manipulate utensils to prepare a sandwich (SAND), and (iv) tool use (TUSE). Main Outcome Measures included self-report of engagement in the activities, improved performance on simulated tasks and observer estimate as measured by time to completion or number of words completed from baseline, correlations among performance measures and self-reports of boredom, neuropsychological symptom inventory (NSI), and The Purdue Peg Motor Test (PPT).

**Results:**

Participants were 19 adults from the community with a 1 year history of non-penetrating traumatic brain injury (TBI) and were able to use computers. Seven had mild, 3 moderate and 9 severe TBIs. Mean score on the Boredom Proneness Scale (BPS): 107 (normal range 81–117); mean NSI:32; mean PPT 54 (normal range for assembly line workers >67). Responses to intervention: 3 (15%)subjects did not repeat all three trials of the tasks; 100% reported they were highly engaged in the interactions; 6 (30%) reported they had a high level of frustration with the tasks, but completed them with short breaks. Performance measures: Comparison of baseline to post training: TOOL time decreased by (mean) 60 sec; SPELL increased by 2.7 words; TUSE time decreased by (mean) 68 sec; and SAND time decreased by (mean) 72 sec. PPT correlated with TOOL (*r*=−0.65, *p*=0.016) and TUSE time (*r*=−0.6, *p*=0.014). SPELL correlated with Boredom score (*r*=0.41, *p*=0.08) and NSI (*r*=−.49, *p*=0.05).

**Conclusion:**

People with chronic TBI of various ages and severity report being engaged in using haptic devices that interact with 3D virtual environments. Haptic devices are able to capture objective data that provide useful information about fine motor and cognitive performance.

## Introduction

Traumatic Brain Injury (TBI) affects 0.4% people in the US and 0.085% are admitted to hospital [1]. Current estimates from the Center for Disease Control (CDC) indicate that at least 1.4 million Americans sustain a TBI annually. TBI affects 475,000 children under age 14 each year in the United States alone. Most are under 45 years of age [2].

This presents a significant challenge for the health care community. Therefore, early detection, proper and timely intervention and followup to prevent secondary disability and assist in societal integration would be desirable [2–4].

The published literature indicates we have recently gained a better understanding of the biology of neurocognitive and neuroaffective deficits associated with TBI [5]. There is acceptance that TBI is both an acute and chronic problem, has a spectrum of severities, varied recovery trajectories and atypical presentations. The variance in presentations and trajectories of recovery, as well as the need for assessments of the effectiveness of treatment requires we use evaluations at multiple time points during recovery that are valid and sensitive. Additionally, the evaluations should include both anatomical and functionally based measures that have real world relevance and address issues that are of concern to individuals.

Current thinking about how to evaluate people with TBI suggests that measures must include complex, sequential and functional assessments [6–8]. This makes evaluation very challenging. Motor function, coordination, apraxia, the inability to sustain attention, minimize distraction and complete tasks in a timely fashion is frequently observed in people with TBI [9, 10]. Additionally, proprioceptive loss and apraxia are likely to influence performance.

Several factors are likely to contribute to normal attention. One, proposed in [11], suggests that this may be seen as two separate processes: one is that it is highly motor driven, repetitive and automatic once learned; the other is that there is a requirement for working memory which includes processing of serial or sequential events. These two processes are likely to take more time for completion and can possibly be differentiated by measuring time to completion and details of the trajectory chosen to reach goals. These may reflect speed of cognitive and information processing [12].

Virtual reality has been applied to both the evaluation and treatment of persons with central nervous system disorders, mainly for stroke [13–15]. The use of simulated environments been used for people with TBI and has helped engage patients in both entertainment and therapeutic activities [16]. Other investigators have used VR in TBI. Some reports address the potential use for problems of attention [17], balance [18] or daily activity skills [19, 20]. The techniques used are most frequently immersive VR with the use of goggles, an approach that presents challenges to some with TBI [17]. These environments have also provided “real” life situations that call for integration of sensory, cognitive and motor activities [21]. Some studies have assessed the potential contributions of this technology to the evaluation and the treatment needs of patients [22].

This development creates an opportunity to use haptic technology for two important clinical applications. The first is the measurement of fine motor movement during functional tasks, which may identify motor strategies to improve function. The second is that haptic technology, which uses force feedback, may be programmed to provide specific trajectories of movement that guides users to task completion, and may positively influence functional outcome either because of its effect on motor planning and performance or process and planning.

One type of virtual reality employs the use of haptic devices that connect the user via touch using force or vibration as feedback. Applications for this technology have been widespread, and include: video games, guided movement for performing surgical procedures, and motion simulation, among others [23–26]. There is an increasing level of interest in this technology because it is engaging, provides objective performance data and permits development of varied and flexible simulated environments [27, 28].

This manuscript reports the results of a pilot study testing the feasibility of using 3D virtual scenes designed to provide motor and visual input for learning.

The hypothesis for this study was that subjects with chronic TBI despite varying severity will report engagement in performing 3D functional simulations using a haptic-computer interface. The primary outcome of this study was the degree to which the subjects were engaged with the haptic using self and observer reports. Secondary outcomes included determination of improvement in performance as measured by time to completion of the simulated motor, process and cognitive tasks. Finally, we wished to determine how the haptic correlates with legacy measures of fine motor and neurobehavioral symptoms.

## Methods

The study was approved by the George Mason University (Mason) Institutional Review Board (IRB) and the National Institutes of Health (NIH) IRB.

Nineteen subjects with non-penetrating mild, moderate or severe TBI, at least one year prior to study, participated. The subjects were recruited via IRB approved flyers posted in the Washington metropolitan area and from addressing TBI support groups directly. Inclusion criteria were: at least 18 years of age, had a non-penetrating traumatic head injury at least 1 year ago, have had experience playing computer games. Exclusion criteria were: unable to read and understand English well enough to complete the written study questionnaires, provide informed consent, hold and move the stylus, if potential participants had an uncontrolled seizure disorder. The subjects visited the institution once and testing and interventions were completed during a single visit.

During testing, the subject sat at a computer terminal with the principal investigator (PI), who remained nearby throughout the trials in order to answer any questions. Team members were present to assist with technical questions, record behaviors, and answer questions asked during the session.

### Study procedure

A brief history and physical exam were performed to assess strength, balance, cognition and coordination. Level of severity severity of initial TBI was assessed by asking the Glasgow Coma Scale rating, if known [29]. Everyone was asked to recall the duration of loss of consciousness, as often this was most frequently known. Medical records were available for confirmation or resolution of difficulty in determining this. The accepted classification is as follows: mild (mental status change or loss of consciousness (LOC) <30 min), moderate (mental status change or LOC 30 min to 6 hr), or severe (mental status change or LOC >6 hr) [30].

All subjects also received the following standardized tests:

1. Boredom Propensity Scale: A boredom inventory was administered to all subjects that asked the subject to describe which behaviors most accurately describe attitudes and behaviors about activities [31].
2. All subjects were asked 4 standard questions about the experience: (i) Did you find the instructions difficult to understand? (ii) Did you find the tasks difficult to perform? (iii) Were you frustrated during the performance? (iv) Did you try to improve your scores? Did you use a strategy to do this?
3. Purdue Pegboard Test: a standardized measure of fine motor proficiency [32].
4. Neurobehavioral Symptom Inventory (NSI): a standard checklist of symptoms frequently experienced by people who have sustained TBI [33].
5. Wolf Motor Function Test (WMFT): a standardized measure of functional fine and gross motor upper extremity activity [34]. The subjects who had no upper extremity symptoms or any neurological deficits were not required to perform the WFMT.
6. All subjects were observed during their testing sessions and an observer recorded spontaneous comments and extraneous movements to better inform the assessment of frustration and engagement.

**Figure 1:**
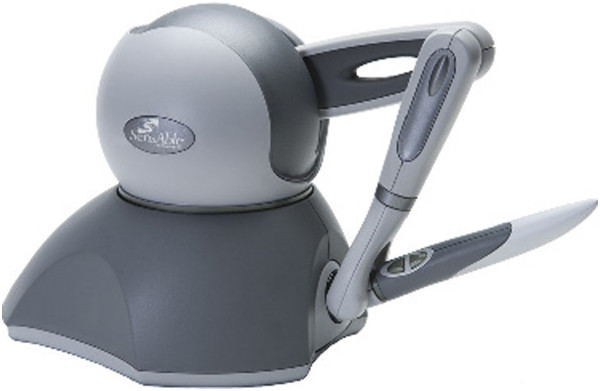
**Phantom®; Omni™.** A haptic device used in our experiments.

The haptic device (see Figure 1) was placed on a table, attached to a monitor. The device is a commercially available, stylus-driven haptic, that manipulates a cursor in the virtual environment. A Phantom®; Omni™ [35] is the haptic interface for the simulations (see Figure 1). It has a small workspace of 160×120×70 *mm*, and it can apply up to 3.3 Newtons (N) of force on its gimbal. It automatically reports the position, velocity and force applied in three-dimensional coordinates.

The subject was seated at an adjustable table, and the stylus was positioned for the left or right hand. No goggles or head gear were used, and the visual image was two dimensional, although the cursor moved in 3D. The room was quiet and lighting was incandescent. The subjects were given breaks as needed to walk, use the restrooms or take refreshment.

The first interaction was a standardized introduction to the haptic. This training program was run to enable the subject to become familiar with how the haptic stylus movement corresponded to the movement of the cursor on the screen and to learn how to grab and manipulate objects in the 3D virtual space.

**Figure 2:**
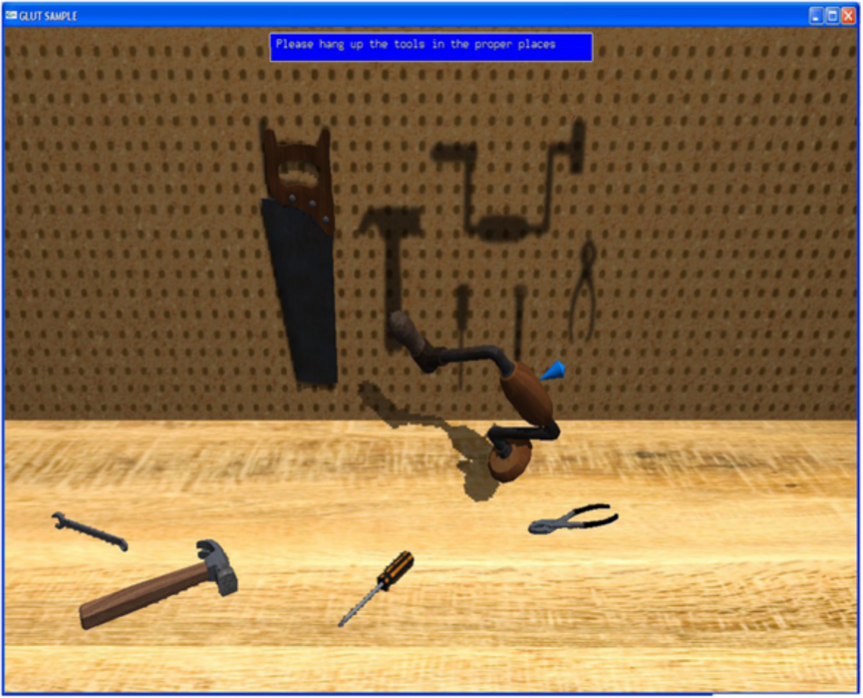
**Workbench cleaning task.** The user needs to pick tools and hang them on the backboard. The tools lock in place when their centroid is close to the center of the shadow.

**Figure 3:**
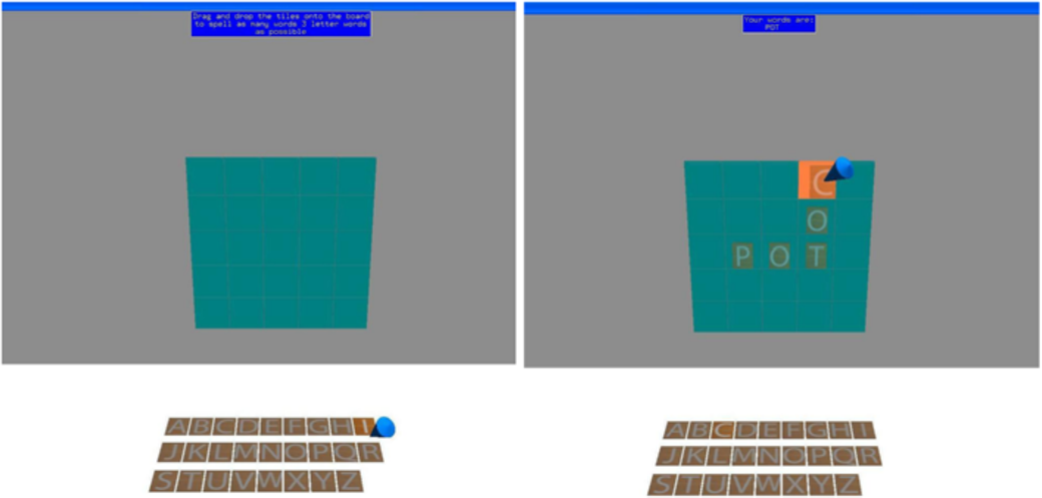
**Forming 3-letter words.** The user needs to spell as many 3-letter (dictionary) words as they can in 2 minutes. The letters can be overlaid on top of other letters. Left: the start state. Right: the user has spelled one word (POT) and is finishing the second (COT).

**Figure 4:**
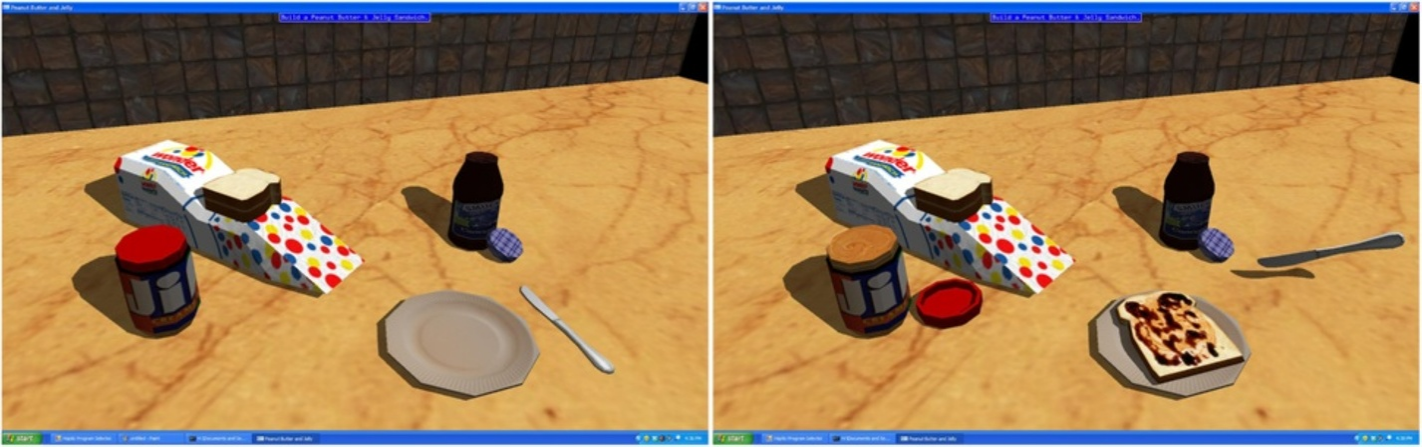
**Sandwich making.** The user needs to make a sandwich. A piece of bread needs to be picked, the peanut butter jar needs to be opened, peanut butter and jelly need to be spread using the knife, and finally a second piece of bread needs to be added. Left: start state. Right: the state before adding the second piece of bread.

**Figure 5:**
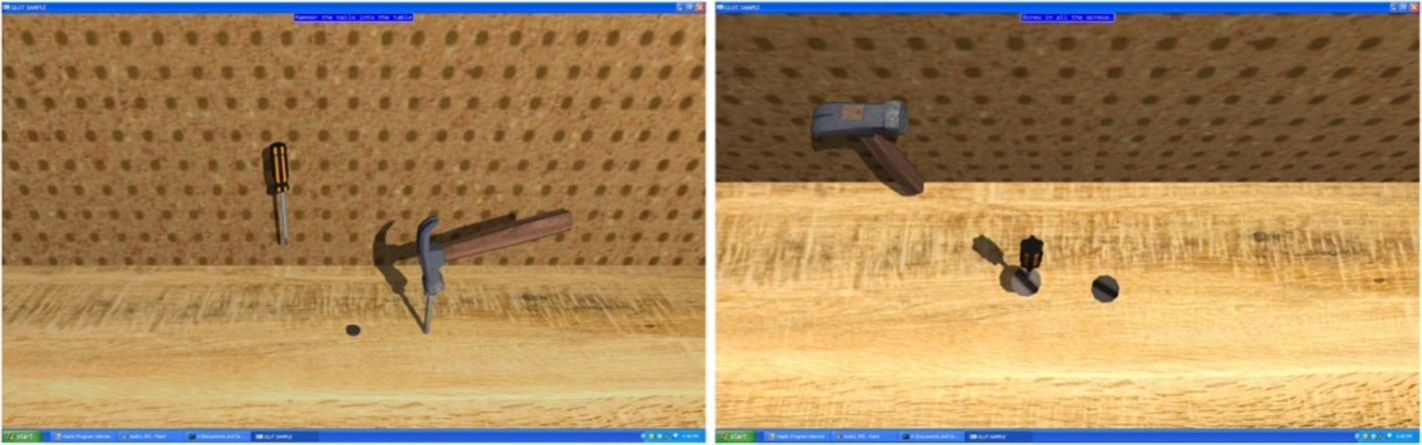
**Tool use.** The user needs to hammer the nails using the hammer and tighten the screws using the screwdriver. Left: hammering nails. Right: tightening screws. The hammer and screwdriver are grabbed by pressing a button the haptic stylus.

Once the introduction was complete, subjects were provided the four virtual scenes in standard order. Each one involved a task to complete. There were 3 trials of each virtual scene, with a time limit of 5 minutes for completion. The word-forming task had to be completed in 2 minutes. In the first trial (see Figure 2) subjects were asked to clear a workbench and mount tools on an upright peg-board wall. The second was (see Figure 3) spelling as many 3-letter words as possible from a set of letter tiles. The third task was preparing a virtual peanut butter and jelly sandwich (see Figure 4). Lastly, the subjects were asked to hammer in two nails and tighten in two screws (see Figure 5). A standard order was selected for convenience.

### Haptic programming

Our approach combined the use of OpenGL programming libraries for graphical presentation, since they provide a set of methods for displaying 3D environments integrating the virtual objects. OpenGL renders 3-D scenes for which visual cues can provide spatial orientation. The program added spatial cues through the implementation of shadow mapping to illustrate the proximity and orientation between objects. OpenHaptics™ was used to program the haptic movement. This is the software used routinely by the haptic manufacturer.

As the user moves the haptic cursor in space, a virtual equivalent is moved on screen in a similar manner, thus providing the user with proprioception. OpenHaptics™ also provides, as a set of features, force-feedback from the haptic cursor as its virtual equivalent touches a virtual object, providing tactile cues from the environment. The OpenHaptics™ libraries provide a means to apply force to the haptic stylus, the pen-like interface that the user is holding, to provide force training in any simulation to our specifications. For auditory cues, OpenAL was integrated into our development framework. OpenAL provides a means for playing sounds as localized in the virtual space, thereby providing spatial cues in terms of volume with sounds further back in the scene being played more softly. The use of sounds can also provide an interaction cue as a sound can be played once an object is touched. Also task feedback can be provided through sound, as either encouragement or suggestions for improvement.

We developed a unique simulation, integrating the different programming libraries in a higher-level programming framework. Included in the development framework is the ability to log any event in a simulation along with the full path that the haptic traverses. The simulation begins with all the tools arranged on the desk table in a specific pattern. The user is told in a text message at the top of the screen to hang the tools in their appropriate locations and that the time to completion will be measured. The back peg board has tool silhouettes drawn to indicate where the tools are to be placed. The user can choose the tool they wish to hang. The tools used in our simulation vary in size and shape and include: hand drill, saw, screwdriver, pliers, and a hammer. The tools were designed to get slightly brighter as the user touches them so that the subject can get a visual cue on top of the tactile cue provided by the haptic. While the users are touching the object they must press and hold the haptic button to grab the object, exactly as in the training simulation.

While holding the tool, they must move it to the appropriate silhouette. Once the centroid of the tool is within a specific Euclidean distance from the center of the silhouette, the tool locks into position, preventing the user from grabbing it again. The four interactive test virtual environments were created using 3D modeling software for constructing the objects. Image manipulation programs were used to construct textures for the objects, using typically familiar virtual objects in real life. The virtual scenes were first tested in subjects without TBI [36] and corrections/refinements were made based on these prior experiences.

We programmed visual cues into the presentations including written instructions, highlighting objects when they were touched and experiencing resistance when the virtual objects were juxtaposed with the wall or surface. One simulation included audio cues that were provided as a tool hit an object. These features were implemented in a development framework for efficient production and modification of realistic, immersive, virtual environments.

### Haptic data capture

Each haptic simulation starts with an automatic calibration of visual and haptic spaces. All procedures were automated. Each subject was allowed 10 minutes for haptic training and maximum 5 minutes per simulation for all other trials. The simulation was stopped either after completion of the task or after 5 minutes. During the computer simulation, all positional and rotational data from the haptic were captured at 1000 Hz. The positional data was captured as (*x*,*y*,*z*) coordinates of the stylus in haptic space—the user feels them at the gimbal of the stylus. The rotational data was captured in the form of quaternions corresponding to the orientation of the stylus in haptic space. Note that with proper calibration—i.e. stylus at neutral position at the beginning of each trial—the haptic/virtual orientation corresponds to stylus orientation in real space. All events corresponding to object touching, un-touching, grabbing and release were recorded with corresponding timing, positional, and rotational information.

In haptic training simulation all events corresponding to object interactions were recorded. The events included touching, grabbing, moving, and releasing objects. In the workbench simulation recorded events include object touching and un-touching, grabbing and release, and release after hanging an object. Note that once an object is hanged it becomes unmovable. In the tool use recorded events include grabbing the tool, tool interaction with nail or screw, movement of nail or screw, and success in completing the task—fully hammering the nail or tightening the screw. In making a sandwich all events such as grabbing an object (e.g. piece of bread or a knife), moving bread, touching the jar cover, touching peanut or jelly or bread with the knife were recorded.

In spelling simulation we counted the number of three letter words completed within the 2 minute allotted time. The recorded events include grabbing and releasing a letter, placing letter on the grid, and completing a three-letter word (checked against a dictionary).

### Statistical analyses

For each of the four tasks—workbench cleaning (TOOL), sandwich making (SAND), word spelling (SPELL), and tool use (TUSE)—we ranked the users by their success in completing the task. For TOOL the completion time was the deciding measure; if the task was not completed the first tiebreaker we used was the number of tools hanged and the second tiebreaker was the time used to hang the tools, i.e. if two subjects hanged the same number of tools, but not all of them, the subjects who used less time for hanging was deemed better. For SAND the completion time was the deciding measure; if the task was not completed the first tiebreaker was the number of completed steps and the second tiebreaker was the time used to complete the finished steps. For SPELL the number of spelled words was the deciding measure; in the case of tie the time used to spell the words was the tiebreaker. Finally, for TUSE the completion time was the deciding measure; if the task was not completed the first tiebreaker used was the number of tightened screws and/or hammered nails and the second tiebreaker was the time used to complete the finished steps.

For all VE tasks we computed Spearman Rank correlation with the standardized tests (Purdue Peg, Neurobehavioral Symptom Inventory, Boredom Scale). The score was computed for both the baseline (first) test and the final (third) test.

## Results

### Subject demographics

Nineteen subjects were consented for this prospective cohort study performed in a biomedical research facility. Demographic and subject group descriptions are presented in Tables 1 and 2. Ten subjects had history of upper extremity functional difficulties but all were able to manipulate the stylus on the computer. Four participants had switched hand dominance since their injury and were performing the computer tasks with their non-dominant hand. Some used both hands during the testing. There were no restrictions placed on the mode of haptic stylus manipulation.

**Table 1:** **Demographic information**

Demographics	Subjects	19				
**Gender**	Male	11	Female	8		
**Age (years)**	Average	50.4	Range	29–78		
**Educational level**	H.S.	7	College	12	Postgrad	7
**Employed before**	Yes	18	No	1		
**Employed afterinjury**	Yes	3	No	15	Student	1
**Marital status**	Single	8	Married	8	Divorced	3
**Parental status**	Parent	5	Grandparent	2	Dep. Child	2
**Independence**	In community	19	Independent	16	Dependent	3

**Table 2:** **Medical status**

Severity of TBI	Severe	9	Moderate	3	Mild	7		
**Glasgow score**	Range	4–14						
**Years from injury**	Mean	10	Range	1–41				
**Hospitalization for**	Neuro. Cond.	13	Rehab.	11				
**Causes of injury**	Fall	9	MVA	7	Blast	2	Sport	1
**Reported pain level**	Severe	6	Moderate	7	Low	6		
**(with usual medic.)**								

Note that severity of TBI is measured using the following criteria. Mild TBI: Brief loss of consciousness, usually a few seconds or minutes; Post-traumatic amnesia (PTA) for less than 1 hour of the TBI; Normal brain imaging results. Moderate TBI: Loss of consciousness for 1–24 hours; PTA for 1–24 hours of the TBI; Abnormal brain imaging results. Severe TBI: Loss of consciousness or coma for more than 24 hours; PTA for more than 24 hours of the TBI; Abnormal brain imaging results.

### Standardized tests: descriptive assessments using self-reports

The scores on the boredom scale are summarized as follows: all 19 reported engagement in the haptic interactions, 17 reported a high level of engagement, 2 reported that they experienced some boredom, though they also stated that they were somewhat engaged. Seventeen finished all 3 trials of each task (89%); 2 finished only one of the three trials for each task (10%), 1 finished the training and all three repetitions of the 3 tasks, except word building, because of low vision. Six reported a high level of frustration (31%), but all completed tasks with short breaks. Self-reports of level of frustration and investigator observed findings reflecting frustration and engagement are summarized in Tables 3 and 4.

**Table 3:** **Engagement questionnaire: perceptions of difficulty, reports of engagement, number of questions**

***Self report***			***Number of subjects***	
Perceived Difficulty with tasks				
Learning and performance both difficult		4		
Learning and performance both easy		3		
Learning easy, performance difficult		11		
Learning difficult, performance easy		1		
Difficulty attributed to lack of ability		11		
Difficulty attributed to equipment		19		
Did you use a strategy to perform the task?	Yes	19	No	0
Were you engaged in the task or bored?	Engaged	19	Bored	2
(Two reported being engaged and bored at times.)				
***Observations***				
Was the subject engaged?	Yes	19	No	0
Was the subject frustrated?	Yes	12	No	5
How many questions did the subjects ask?	Mean	39.3	Std. dev.	20.3

**Table 4:** **Reactions and responses: spontaneous subject comments**

*Mean number of counted questions and remarks by subject*	34.4 *(Std. dev. 17.1)*
*Mean number of counted coaching remarks by researchers*	34.1 *(Std. dev. 21.1)*
*Opinions spontaneously expressed by subjects, classified by type, ranked by frequency*	
Frustration	81
Information seeking/confirming	56
Self-description	39
Strategies	36
Recommendations with respect to	35
tasks and experience	
Achievement/Satisfaction	34
Engagement	15
Anxiety	9
Boredom	6
Fatigue	5
*Frequency of comments per task*	
General comments on project (excluding recommendations)	77
Hammer and Screwdriver	50
Training	44
Workbench Cleaning	42
PB&J Sandwich	39
Recommendations	35
Word Building	29

Scores of the Boredom Propensity Scale inversely correlated to age and education level (*r*=0.453 and *r*=0.416, respectively, *p*=0.05 and 0.076 respectively). Neither NSI or PPT showed significant correlations with the boredom measure. Baseline measures for SAND moderately correlated with education level (*r*=0.433; *p*=0.06). TOOL moderately correlated with age (*r*=0.51; *p*=0.07).

Results of the scores received by subjects on standard tests are presented with means and standard deviations. These were compared to normal data in Table 5. A total of 10/19 (52%) were thought to have some upper extremity deficit. Each of those was administered the WFMT and all completed the test. Correlations between haptic performance and standardized testing are presented in Table 6.

**Table 5:** **Performance on standardized tests**

***Standardized Test***	***Mean***	***Std. Dev.***	***Normal***	***Std. Dev.***
Purdue Pegboard	54	15.3	>67	
Test (PPT)				
Boredom Propensity	180	17.7	81–117	
Scale (BPS)				
Neurobehavioral Symptom	33	26.2		
Inventory (NSI)				
Wolf Motor Function	1.7	.49	1.2	0.2
Test (WMFT)				

Normal ranges are not available for the Neurobehavioral Symptom Inventory (NSI) [33]. Normal mean for WMFT is 1.2 with a std. dev. 0.2. WMFT was taken by 10 of the 19 subjects.

**Table 6:** **Correlation between haptic performance and standardized testing**

***Time for Workbench Clearance (TOOL)***				
***Standardized test***		TOOL baseline		TOOL 3rd trial
	*ρ*	*p*-val	*ρ*	*p*-val
NSI	0.0676	0.7833	-0.1429	0.6428
Boredom	0.2993	0.2133	0.2418	0.4258
Purdue	-0.326	0.1732	-0.652	**0.0157**
***Hammer and Nail Completion Time (TUSE)***				
***Standardized test***		**TUSE baseline**		**TUSE 3rd trial**
	*ρ*	*p*-val	*ρ*	*p*-val
NSI	-0.3186	0.1838	-0.0647	0.8118
Boredom	-0.1930	0.4286	-0.0441	0.8711
Purdue	-0.5191	**0.0228**	-0.5984	**0.0143**
***Number of 3 Letter Words Completed in 2 Minutes (SPELL)***				
***Standardized test***		**SPELL baseline**		**SPELL 3rd trial**
	*ρ*	*p*-val	*ρ*	*p*-val
NSI	0.0412	0.8669	-0.4941	**0.0517**
Boredom	0.4070	0.0837	0.0735	0.7867
Purdue	-0.3373	0.1579	-0.3906	0.1347

There is a statistically significant correlation between the having high score (high dexterity) for the Purdue Peg Test and time for completion of the tool clearance for the 3rd trial; and hammering the nail and screwing the screw for the first and third trials. There is a statistically significant correlation between number of words formed and a low number of symptoms on the NSI.

Intra-subject change for variables of interest related to word formation was tracked for the three trials (see Table 7). The table includes a count of the number of tiles manipulated to form each 3 letter word, the number of words formed and the number of tiles manipulated per word formed for each subject. Improvement in performance was defined as the increase in number of words formed per trial. Variables contributing to the increased word formation included changes with repeated trials of the ratio of the length of the trajectory of the stylus/distance traveled, mean time it took to grab the tile, number of tiles touched/number of words formed. Analyses were performed on changes in the amount of time transpired to approach the tile, the trajectory of the stylus to reach the tile and the number of tiles touched. Table 8 presents the correlations between the number of words formed and the variables listed when comparing the first and the third trials. Number of tiles/word formed correlated negatively with performance (number of words formed in 2 minutes) at both the first and third trials. The most significant correlations associated with the improvement in number of words formed are the decrease in mean time spent on each tile and the efficiency (ratio of the trajectory and the distance traveled). The greatest performance improvement occurred when there was a reduction in time spent with the stylus on the tile.

**Figure 6:**
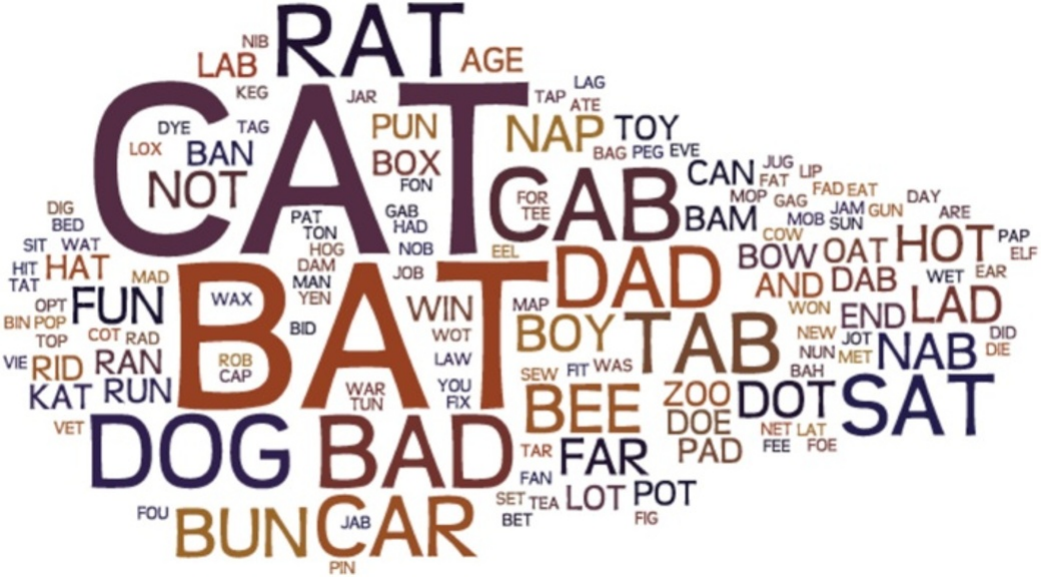
**Word cloud.** There were 264 words, 150 unique. The most frequently spelled words were CAT (14) and BAT (12). All other words appear 6 or fewer times. 100 words appeared once and 28 words appeared twice. The size of each word in the figure is proportional to its frequency.

The word forming data showed that there were 264 words, 150 unique. The most frequently spelled words were CAT (14) and BAT (12). All other words appear 6 or fewer times. 100 words appeared once and 28 words appeared twice. Applying the world cloud technique, we show the pictorial representation of this frequency in Figure 6. Repeating the same word in multiple trials is associated with reducing the number of touches to different tiles.

**Table 7:** **Variables of interest pertaining to tile manipulation and word forming per individual subject for each of three trials**

		***Number of tiles***			***Number of words***			***Tiles/Word***	
**Subject**	**T1**	**T2**	**T3**	**T1**	**T2**	**T3**	**T1**	**T2**	**T3**
1	13	22	19	3	6	13	4.33	3.67	1.46
2	9	17	22	1	4	4	9	4.25	5.5
3	13	10	15	3	4	5	4.33	2.5	3
4	16	–	–	4	–	–	4	–	–
5	13	14	11	1	2	2	13	7	5.5
6	22	24	27	6	5	9	3.67	4.8	3
7	20	–	–	8	–	–	2.5	–	–
8	20	19	24	6	6	8	3.33	3.17	3
9	30	33	37	8	9	11	3.75	3.67	3.36
10	13	13	12	4	4	3	3.25	3.25	4
11	5	–	–	0	–	–	*∞*	–	–
12	20	23	13	3	3	3	6.67	7.67	4.33
13	12	24	18	3	3	5	4	8	3.6
14	16	20	25	5	7	13	3.2	2.86	1.92
15	17	12	16	1	2	4	17	6	4
16	13	11	14	4	5	5	3.25	2.2	2.8
17	7	7	17	2	2	4	3.5	3.5	4.25
18	25	30	37	10	10	13	2.5	3	2.85
19	16	17	17	6	6	7	2.67	2.83	2.43

Variables of interest include: number of tiles manipulated, number of dictionary words formed and average number of manipulated tiles per dictionary word formed. A dash represents an incomplete trial, T1 =trial 1, T2 =trial 2, and T3 =trial 3.

**Table 8:** **Spearman correlations for tile manipulation and word forming variables with the number of words formed**

***Variable***	***Outcome***	***ρ***	***p***
# of tiles per word 1st	# of words 1st	−0.84	7.18*e*−06
# of tiles per word 3rd	# of words 3rd	−0.84	5.17*e*−05
Mean time on tile 1st	# of words 1st	−0.47	0.04
Mean time on tile 3rd	# of words 3rd	−0.80	1.96*e*−04
Distance ratio to tile 1st	# of words 3rd	−0.32	0.191
Distance ratio to tile 3rd	# of words 3rd	−0.6	0.014

Data are reported for change in variables from first to third trials with respect to total words formed in 2 minutes. The time is measured in seconds and the distance is measured in millimeters.

Intra-subject change for variables of interest related to clearing tools from the workbench and placing it on the pegboard was tracked for each of the three trials are shown in Table 9. Improved performance was defined as the number of tools cleared per minute per trial. Variables contributing to the improved time in clearing the workbench are presented in Table 10. These included # of touches/tool, time it took to grab and place the tool, and the distance the stylus traveled to grab and place the tool. The average time taken per tool grabbed and placed captures the relative performance rank for each subject. The mean time to grab a tool and the mean distance to grab a tool correlate with performance in the first and third trials. Significant improvement occurs when there is a decrease in number of touches and the amount of time taken and the distance travelled to place a tool.

**Table 9:** **Variables pertaining to tool manipulation presented by individual subject for each of three trials**

		***# of tools placed***			***# of tool touches***			***Total time***			***Time/tool-placed***	
**Subj.**	**T1**	**T2**	**T3**	**T1**	**T2**	**T3**	**T1**	**T2**	**T3**	**T1**	**T2**	**T3**
1	6	5	6	88	33	11	831.2	546.2	203.7	138.5	109.2	33.9
2	0	4	–	0	38	–	300.0	300.0	–	*∞*	75.0	–
3	0	5	5	0	20	34	300.0	185.7	217.5	*∞*	37.1	43.5
4	4	–	–	58	–	–	300.0	–	–	75.0	–	–
5	0	3	5	0	91	36	300.0	300.0	204.2	*∞*	100.0	40.8
6	5	5	5	16	10	16	115.2	78.1	65.5	23.0	15.6	13.1
7	4	–	–	103	–	–	300.0	–	–	75.0	–	–
8	4	0	3	61	0	75	300.0	300.0	300.0	75.0	*∞*	100.0
9	5	5	5	23	9	15	160.4	81.5	64.2	32.1	16.3	12.8
10	4	1	4	19	31	51	300.0	300.0	300.0	75.0	300.0	75.0
11	4	5	5	36	19	33	300.0	120.6	163.5	75.0	24.1	32.7
12	3	4	5	60	88	28	300.0	300.0	101.8	100.0	75.0	20.4
13	1	2	5	37	9	30	300.0	300.0	255.3	300.0	150.0	51.1
14	5	5	5	49	19	52	197.9	143.1	249.0	39.6	28.6	49.8
15	5	5	5	13	13	24	137.7	72.3	127.3	27.5	14.5	25.5
16	3	3	3	73	59	45	300.0	300.0	300.0	100.0	100.0	100.0
17	2	4	3	8	36	29	300.0	300.0	300.0	150.0	75.0	100.0
18	5	5	5	66	30	16	222.5	101.9	58.6	44.5	20.4	11.7
19	0	2	4	0	42	36	300.0	300.0	300.0	*∞*	150.0	75.0

Variables of interest include: number of placed tools, number of tool touches, total time used in trials, time per tool placed in each trial. A dash represents an incomplete trial, T1 =trial 1, T2 =trial 2, and T3 =trial 3. The time is measured in seconds and the distance is measured in millimeters. Note that three subjects did not complete all trials.

**Table 10:** **Spearman correlations for various workbench measures**

***Variable***	***Outcome***	***ρ***	***p***
# of tool touches 1st	Time per tool placed 1st	−0.37	0.12
# of tool touches 3rd	Time per tool placed 3rd	0.74	0.001
Mean time to grab tool 1st	Time per tool placed 1st	0.99	1.64*e*−15
Mean time to grab tool 3rd	Time per tool placed 3rd	0.88	7.67*e*−06
Mean time to place tool 1st	Time per tool placed 1st	−0.13	0.60
Mean time to place tool 3rd	Time per tool placed 3rd	0.92	3.21*e*−07
Mean distance to grab tool 1st	Time per tool placed 1st	0.97	2.21*e*−11
Mean distance to grab tool 3rd	Time per tool placed 3rd	0.86	1.89*e*−05
Mean distance to place tool 1st	Time per tool placed 1st	−0.18	0.46
Mean distance to place tool 3rd	Time per tool placed 3rd	0.80	1.80*e*−04

Data are reported for change in variables from first to third trials with respect to time per tool placed. Variables of interest with respect to time taken per tool placed: number of tool touches, mean time taken to grab and place a tool, mean distance travelled to grab and place a tool. The time is measured in seconds and the distance is measured in millimeters.

## Discussion

The past decade has witnessed an increasing interest in the application of new technology to problems facing people with disability. One application has been the use of robotic devices, virtual reality (VR) and telemedicine. Investigators have reported the feasibility and utility of robotics and VR for people who have suffered stroke [14, 15, 24], movement disorders [13], TBI and PTSD [16–20, 22]. These studies usually utilize games, adapted for possible therapeutic purposes, immersive environments and repetitive, motor exercise. Each has a potential contribution to make, but differs significantly from the application we explored in this clinical feasibility study.

This study was designed to determine whether subjects who had had TBI at least a year prior to study entry were both interested in, and able to manipulate a pen-like stylus in simulated 3D environments that had functional relevance. The instrumentation, software and study design were developed to test several concepts. Would subjects with chronic TBI engage in a repetitious, simulated functional task? The model for the simulations was a frequently used, labor intensive evaluation, the Assessment of Motor and Process Skills [37, 38]. To the best of our knowledge, use of a hand held device, navigating 3D space to perform functional tasks has not previously been reported. Second, would this type of intervention be suitable for subjects with a broad spectrum of severities? It is worthwhile to learn whether this approach and technology has utility for those with mild as well as severe TBI. The former group might be bored and unchallenged. The latter might be unable to focus and participate. Feasibility studies can benefit from using a broad based population. Clinical trials, on the other hand, need more homogeneous populations. Third, we wished to determine whether the haptic device could capture temporal and spatial data during the subjects’ interaction with virtual space and whether these data could be correlated with an arbitrary performance measure, such as speed to task completion or number of words formed.

The interactions required motor coordination, motor processing, attention and patience, hence engagement was the primary outcome. Engagement was measured using a standard self-report and a standardized, real-time observational, descriptive assessment of engagement, frustration and boredom recorded by the investigators. Both are presented in Table 3. The boredom questionnaire has not been validated in this population, but it was coupled with standard questions about boredom, frustration and engagement and direct observation and scoring of subjects by investigators who were present throughout the trials. All subjects reported that they were engaged in the activities, which was corroborated by the independent observations of the research team. Not all were engaged to the same degree.

In our previous work [36], a similar approach was taken for a cohort of 21 healthy college students. The mean self-reports were higher for engagement and lower for frustration and boredom than the group with a history of TBI.

Despite the self-report and observational corroboration, not all subjects completed all tasks. We attributed this to frustration voiced by several individuals in the study group. Some of this frustration resulted from difficulty manipulating the haptic, which is the size of a fat pen and has only 270 degrees of rotation. This was the most frequent complaint. Some subjects had difficulty managing the cursor in 3D, could not decipher where it was with respect to the object to which they were moving and were unable to utilize cues (shadow, resistance at surface interfaces, highlighting objects) to assist in solving this difficulty. Objectively, we were able to document that many who voiced frustration did not maintain the attachment of the stylus to the virtual object. In general the usage of the hammer/screwdriver was perceived as most challenging and had the most comments pertaining to the task. Adding sound to the hammer and screwdriver contacting the nail/screw, did not seem to mitigate the frustration.

Despite frustration with instrumentation, subjects showed improved performance. We defined performance as a decrease in the amount of time to completion of tasks when comparing the first to a subsequent trial for each of the virtual scenes. This finding is highly suggestive that subjects were engaged and were motivated to improve their performance. Additionally, in real space, speed to completion of tasks has been shown to correlate positively with functional outcomes in people who have sustained TBI [39]. In the referenced work, it was correlated with performance on the PPT. Our performance measure was time to completion and for the the fine motor activities correlated with this test.

Our interpretation was that improvement occurred over the repeated encounters, although not all participants showed the same degree of improvement. Improvement in performance time did not correlate with severity of TBI, time spent in rehabilitation, number of injuries, level of education, or age. The data collected from the haptic showed that decreased time to completion was a function of how long it took to get to the object and this was related to the path selected. The correlations between number of tools placed and selected variables of interest show that the average time taken to grab a tool and place it describes the rank for each subject. Additionally, there is significant improvement in the correlations between the first and third trials with respect to mean time on the tool and distance the tool travels to reach the mounting board.

Word forming was experienced as the least frustrating task and was associated with the fewest comments about the instrumentation. Comments made referred to the size of the tiles and relative lack of contrast between letters and background. Despite the relative ease of tile manipulation, most subjects did not utilize spatial strategies to improve their scores. By strategy, we mean a plan of action designed to achieve an aim. Most participants when asked replied that they used a strategy to approach and move objects. However, this was only occasionally articulated. For example, although many chose to follow a rhyming technique for word selection (‘cat’, ‘bat’, ‘rat’, ‘sat’, etc.) only one subject literally swapped out a single letter and this strategy was the most successful. The most successful in increasing the number of words formed did so by using the fewest number of tiles/word. This was a function of changing few letters for each new word and spending relatively little amount of time on the tile.

Our analyses enabled us to map the word selection and deduce selection patterns in the word forming task. These included mainly rhyming and alliteration as being successful strategies for prompting the next choice and thereby increasing numbers of words. However, it was associated with a decrease in time on tiles. The correlations suggest that increase in number of words spelled is associated with decrease in the amount of time spent on each tile. There are many possible explanations for this decrease with practice. Individuals may have become more comfortable with the stylus, have developed a strategy of letter selection that is efficient using rhyming as a prompt and therefore having to switch only 1 letter (i.e. cat/rat/bat/sat/ etc.) or developed a smooth motor trajectory that moves the stylus in a direct path. The most significant finding is the change in the correlation between number of words formed and the mean time on the tile. This change between the first and third trials suggests that subjects who spent less time on the tiles may have developed a strategy to do this.

Almost all participants indicated upon questioning that they had devised a strategy to help improve performance and/or deal with frustration. For example, some kept tapping their feet or talking though the experience and coached themselves. Others indicated that a shadow, which was programmed as a positional cue, helped with 3D positioning in virtual space. Not all were able to articulate what the strategy was. Strategies varied from person to person, but the entire cohort indicated that they were aware of certain difficulties and tried to learn from their prior trial experiences to improve upon their performance. The ability to strategize may be an important link to our understanding brain plasticity and learning. The willingness to strategize may be an important indicator of motivation and may help in recovery [40, 41].

The group as a whole had mean performance scores on the Purdue Pegboard Test significantly below the mean for factory workers involved in manual tasks (the population used to establish norms). The motor tasks (clearing the workbench and the hammer/nail functional task) correlated with the Purdue Pegboard Test scores, providing validation of the virtual tasks with a fine motor task. This does not establish the validity of a virtual task with respect to function or carryover into real activities. While we observed improved performance in the virtual task, utility in real life and its relevance to specific functional tasks remains to be tested. However, the correlations we demonstrate between virtual performance and performance on standardized tests used to measure symptom burden (NSI) and fine motor (Purdue Peg) encourages this type of testing in the future.

Word-forming ability did not correlate with this test, but did correlate with the NSI. These findings suggest that for fine motor tasks a motor performance measure is suitable. For a cognitive task, symptom burden best correlates with a task that has neurocognitive requirements.

The NSI is a measure of neurobehavioral symptom burden in people who have had TBI [33], and is frequently used in the military and veteran populations. The NSI score has been reported to correlate with severity of TBI [42] and has been recommended for use in the Common Data Elements, an interdisciplinary effort to reach consensus about standardizing the approach to evaluation for people who have sustained TBI [43, 44]. The mean scores for the NSI in the cohort in this study were lower than the non-TBI population. The NSI is a reliable and valid measure of post-TBI and concussive symptoms, it is influenced by post-traumatic stress, anxiety and depression [45]. We did not control for these symptoms in our study.

The data captured by the haptic are objective and sensitive. We are able to demonstrate that improved task performance (increase in total number of words formed or number of tools cleared from the workbench) occurs with repetition of the task. The haptic permits measurement of temporal and spatial data when analyzed in terms of discreet events, for example, distance traveled to attach letter tile or tool, time on tool or letter, number of manipulations of tool or letter; and amount of time to task completion. Using temporal and spatial variables we correlated them with number of words spelled and number of tools cleared from the workbench. Most subjects improved their performance after the 3 trials.

One of the initial thoughts when designing this study was that an individual’s age and level of education would influence outcomes. Our research team discussed the possibility that older people would perform less well, in part because of their relative lack of familiarity with computers and haptic (video game) technology. We also considered level of education as a variable that might negatively influence learning. Neither is the case in this small pilot study. Motor performance was, in general, more likely to correlate with PPT scores and the word forming to some extent, to neurobehavioral symptoms and boredom measures.

This study was designed to assess engagement of participants in simulated tasks in order to answer questions about feasibility of using haptic technology in clinical settings. It has several limitations. In general, people with TBI may experience difficulty with attention to task and focus. This attention deficit may reduce the ability to perform both motor and cognitive tasks. We did not measure attention to task, and are aware that this may be a study limitation. Nonetheless, only 2 subjects chose not to complete all 3 trials for each task, and the great majority of participants demonstrated significant improvement in performance measures. Additionally, the number of subjects studied was small, was cross sectional, and was quite heterogeneous. Age range was more than 50 years, the time between injury and study varied widely. The nature of the injury varied from blast, to sports concussions; and subjects varied considerably in their clinical severity and their post-injury treatments ranging from no treatment to inpatient rehabilitation. Hence, the observations reported cannot be targeted to a particular post-TBI patient profile. The data collected were historical and were obtained mainly from subject recall which may be inaccurate. We did not think this would compromise the findings, and helped assure us that this technology may be used in a heterogeneous population, similar to what is seen in clinical practice.

The design consisted of a single visit to an institution that lasted multiple hours for evaluation and testing which may have been more fatiguing to some than others. There were no follow-up appointments to assess whether newly acquired “skills” or the improved performance we observed persisted. Lastly, while the group did demonstrate a positive change in the time to completion or number of words completed in the simulated environments, we have no data about carryover into functional activities in non-simulated environments. These preliminary data are encouraging for further, longer term trials to assess the role haptics may play as evaluation and possible therapeutic interventions.

## Conclusion

We report the results of a study demonstrating that a group of research subjects who had TBI more than 1 year prior, engaged in interactive virtual 3D fine motor and cognitive tasks using a haptic device. The subjects completed the tasks, demonstrated improvement in performance over baseline with a high level of interest and engagement.

Significant correlations were seen between fine motor standardized testing (Purdue Peg) and motor performance in virtual space and neurobehavioral symptoms and word forming performance. The haptic device was able to record objective temporal and spatial data and may be useful in evaluating motor performance in people with TBI. Utility as a diagnostic tool and an outcome measure requires a properly sized, prospective, controlled clinical trial.

Haptic technology should be further explored to determine effectiveness in the treatment of TBI related disability.
